# Rapid design and implementation of a UVC decontamination room

**DOI:** 10.1038/s41598-022-04926-4

**Published:** 2022-01-17

**Authors:** Andrew Hummel, Awatef Ergai, LeeAnna Spiva, Sharlene Toney, Austin Crawford

**Affiliations:** 1 Kennesaw State University, 1100 South Marietta Pkwy, Marietta, GA 30060 USA; 2 Wellstar Health System, 2000 South Park Place, Atlanta, GA 30064 USA

**Keywords:** Occupational health, Disease prevention

## Abstract

With the recent COVID-19 pandemic that has swept the world and the nation, hospitals around the country have experienced shortages in Personal Protective Equipment, specifically N95 filter face-mask respirators (FFRs). This has created the need for facilities to develop sterilization processes to enable reuse of face masks by the health care personnel. Among the various methods of sterilization, UVC light exposure is the easiest to implement given the factors of time, safety, and availability. Face masks and/or other PPE are exposed to UVC light for a specified time to kill any viruses or bacteria that may reside on the surfaces of the masks. A collaborative effort was formed in April of 2020 between Wellstar Health System and Kennesaw State University to (1) setup an appropriate sterilization room at a Wellstar hospital (2) develop the procedural guidelines necessary to ensure quality control and (3) assess employees’ perceptions of the N95 FFR decontamination process and efficacy. This paper will first describe the methodology used to validate the layout of the room, which consists of a rudimentary analytical analysis of the UVC photon intensity from bulb-to-mask, computer simulations to determine the lighting power density throughout the room, and experimental measurements to confirm the appropriate energy deposition. This paper will then document the procedures for handling and processing the pre- and post-sterilized masks followed by employee survey findings. It is the hope of the authors that this paper will serve to provide a generic blueprint for hospitals and other organizations to follow if a future need arises for rapid UVC decontamination.

## Introduction

The COVID-19 pandemic, with its exponential spread of the coronavirus, has impacted healthcare systems worldwide, and healthcare workers and professionals are at increased risk due to their close interactions with infected patients. The instant demand for personal protective equipment (PPE) worldwide has led to shortages in PPE, especially N95 Filtering Facepiece Respirators (FFRs), the primary barrier to mitigate disease spread. The high demand for N95 (FFRs) cannot be met by the global supply chain, increasing production, nor decreasing their use^1^, potentially putting healthcare workers and professionals at increased exposure risk.

In specific emergencies, a possible strategy during PPE shortage is to reuse FFRs after an effective biologic decontamination process. The Centers for Disease Control and Prevention^2^ reports that Ultraviolet-C germicidal irradiation (UVGI), vaporous hydrogen peroxide, and moist heat are the most promising methods to decontaminate FFRs to maintain the availability of FFRs during capacity crises.

All material or bodies, whether alive or inanimate, that possess a temperature greater than absolute zero continuously emit thermal radiation energy in the form of photons. This radiation arises due to energy transitions of atoms, molecules, and electrons, and the amount of radiation emitted increases as the temperature of the body increases^3^. This thermal energy is emitted over a spectrum, and the most obvious, and important, is the part of the spectrum constituting visible light which comes from the sun. However, ultraviolet (UV) light is also emitted by the sun and can be artificially produced via fluorescent lamps operating at sufficiently high temperatures. The only difference between UV light and visible light is the fact that the photons are emitted at a higher frequency, and hence, a higher energy. The UV spectrum can be further divided into type A, B, or C, where again the only difference is the energy of the photons (with C designating the higher energy). It is this high energy UVC radiation that is used to kill or denature various bacteria and viruses, and it is well known that an exposure of only several milli joule per square centimeter (about 5 mJ/cm^2^) is needed to achieve the desired sterilization effect^4^. However, since all viruses are different and little was known about the unique susceptibility of the SARS-CoV-2 virus (COVID-19), a conservative value of 60 mJ/cm^2^ was the target exposure to ensure a wide margin of safety. Other studies^5–7^ have sought an exposure value closer to 1000 mJ/cm^2^, but this high exposure was deemed unnecessary since a viral reduction of 99% was observed for all samples examined by Tseng and Li at a maximum exposure of about 17 mJ/cm^2^^4^.

Moreover, the structural integrity of the FFRs material after UVGI treatment is critical to the reuse of the FFR. If structural integrity is compromised, it degrades the FFRs filtering power against the respiratory infectious particles and may release toxic or irritating residues on the respirator surface^8^, placing healthcare workers at increased risk of exposure. FFRs reuse can be validated by the original healthcare worker conducting an integrity test including seal (fit check). We report here on a locally implemented UVGI room for FFR decontamination at our institution and efforts to ensure that every mask placed throughout the sterilization room would receive the appropriate exposure, 60 mJ/cm^2^. In addition, we report on quality assurance measures, qualitative and quantitative, to ensure the structural integrity FFRs after UVGI treatment.

## Room design

### Analytical assessment of the photon exposure

Although UVC radiation spans a spectrum of wavelengths ranging from approximately 0.01 – 0.40 µm, the most readily available bulbs emit at the 0.254 µm wavelength. It should be noted that there are other wavelengths that are known to be effective (i.e., strongly absorbed) such as 0.222 µm^9^ since DNA molecules will absorb radiation up to a maximum wavelength of 260 nm^10^. Thus, the energy associated with photons of the 0.254 µm wavelength can readily be determined using the simple relation developed by Max Planck below relating the photon energy to the frequency^11^.

$$E = h\nu = h\frac{c}{\lambda } = \left( {6.1260693*10^{ - 34} \;{\text{J}}\;{\text{s}}} \right)\left[ {\frac{{3.0*10^{8} \;{\text{m/s}}}}{{2.54*10^{ - 7} \;{\text{m}}}}} \right] = 7.236*10^{ - 19} \;{\text{J}}\;{\text{per}}\;{\text{photon}}$$

To determine the exposure, one ultimately needs the number of photons emitted per second from the UV source (a fluorescent bulb in our case). Since each bulb used was rated to emit 48 watts (W) of UVC light, and 1 W equals 1 J per second, one can divide this by the energy of each photon to get the total source strength, $S$.

$$S = \frac{{48\;{\text{J/s}}}}{{7.236*10^{ - 19} \;{\text{J/photon}}}} = 6.634*10^{19} \;{\text{photons/s}}$$

**Figure 1:**
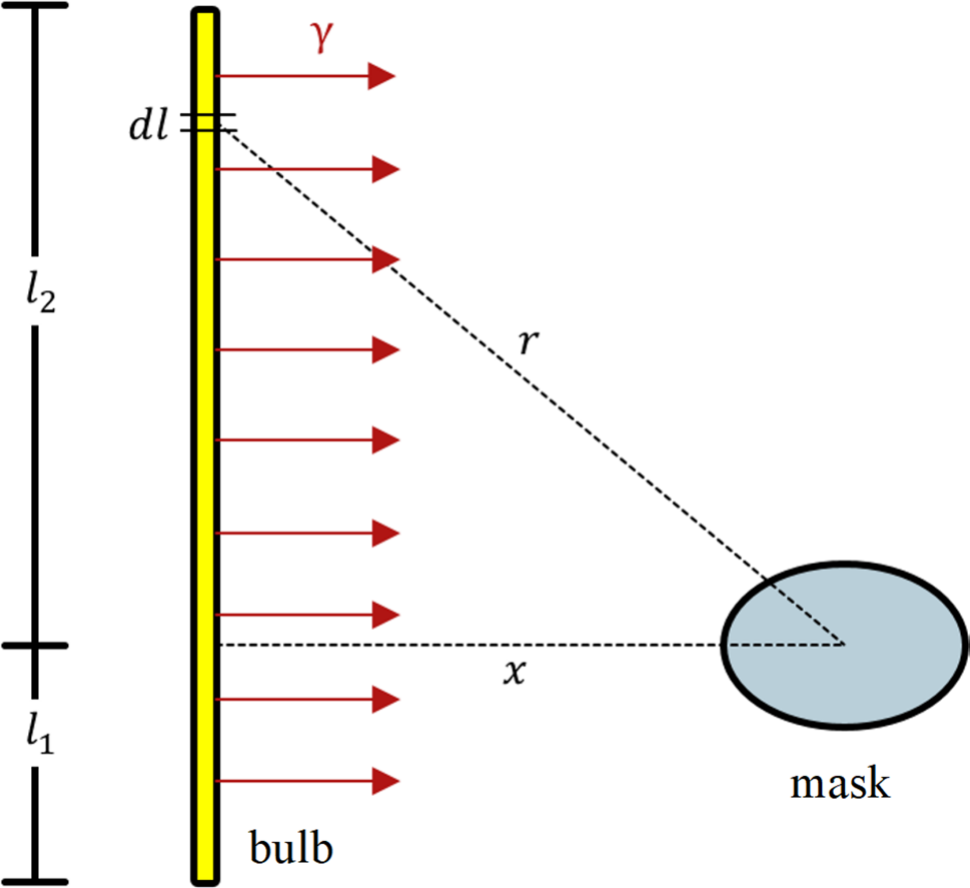
Approximate geometric orientation between the UV bulbs and masks.

Although we now have the total number of photons emitted, we must account for the geometry of the source (bulb) as well as the geometric orientation between the source and the targets (masks) to determine the number of photons that will actually strike the masks and become absorbed (deposit energy). This is shown schematically in Fig. 1. Each bulb measures 5-ft long and is therefore best approximated as a finite line source. If we assume that the UV light emitted from each bulb is isotropic or equal in all directions, then we can use the equation below to yield the number of photons that would strike a target (mask) at some distance $x$ away[^11^, p. 340].

$$\phi = \frac{{S_{l} }}{4\pi x}\left[ {\tan^{ - 1} \left( {\frac{{l_{2} }}{x}} \right) + \tan^{ - 1} \left( {\frac{{l_{1} }}{x}} \right)} \right] \to \frac{{{\text{photons}}}}{{{\text{cm}}^{2} \;{\text{s}}}}$$

In the equation above, $S_{l}$ is simply the source strength, $S$, divided by the total length of the bulb, $l$. This flux quantity, $\phi$, is then determined by assuming that the masks to be irradiated lie along the midplane of the bulbs and that the distance between the bulbs and masks is 8 ft, which is the rough midpoint distance in the sterilization room. Dividing this quantity by the energy of each photon that was previously determined will yield the units of exposure that we desire.

$${\text{exposure}} = \frac{\phi }{E} \to \frac{{{\text{mJ}}}}{{{\text{cm}}^{2} }}$$

For a single bulb that measures 5-ft long rated at 48 W, it will take approximately 16 min to achieve the desired exposure goal of 60 mJ/cm^2^ if the mask is placed 8-ft away along the bulb centerline. If two additional bulbs are accounted for at distances of 10-ft and 12-ft from the mask, the irradiation time drops to about 7.65 min.

### Room layout and experimental measurements of the photon exposure

**Figure 2:**
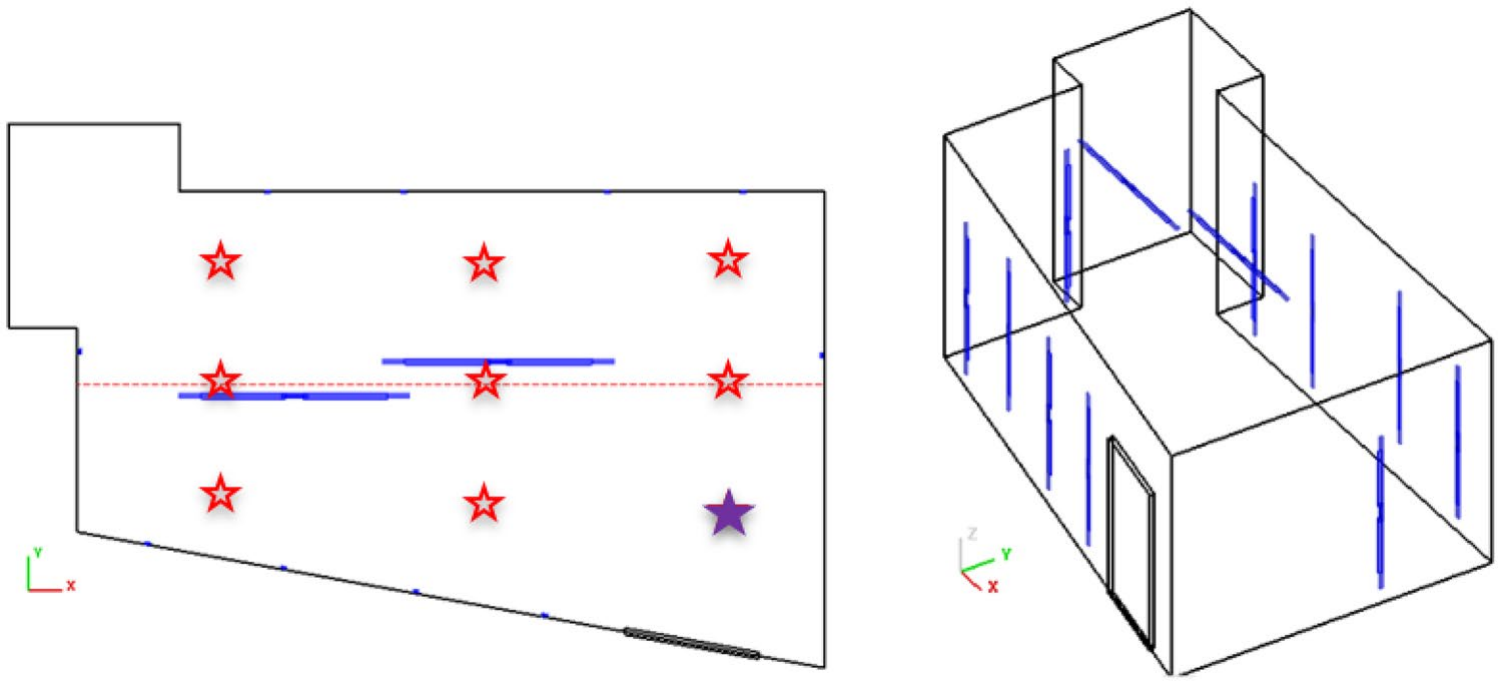
A top-down (left) and isometric (right) view of the UV sterilization room with bulb placements.

**Figure 3:**
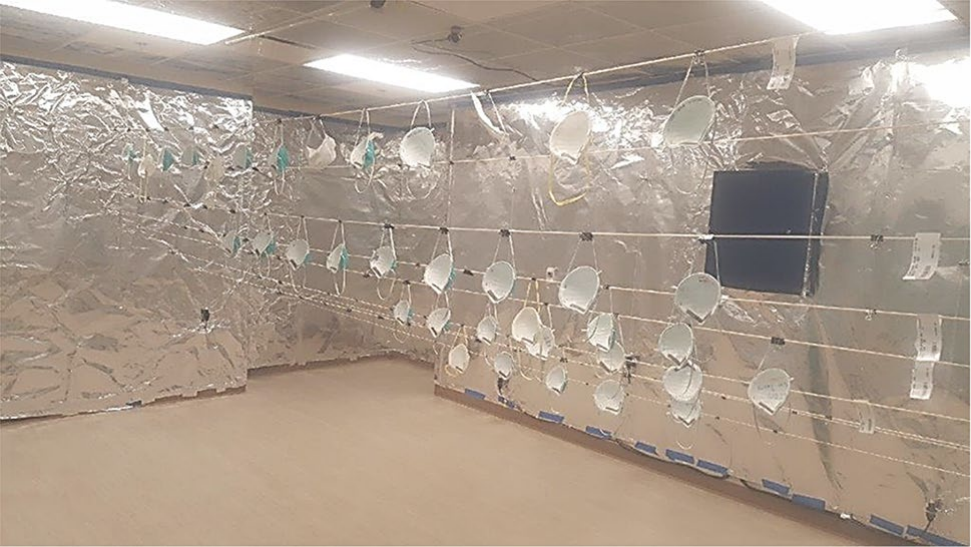
Picture taken of the sterilization room at hospital. The walls are covered with aluminum foil, and facemasks are strung on several different lines across the room.

A total of twelve 5-ft long bulbs were placed around the sterilization room as shown in Fig. 2. Four bulbs are on the front wall with door, four bulbs are on the back wall, two bulbs are on the ceiling, and one bulb each is on the remaining walls. All walls have been covered with standard aluminum foil due to the highly reflective property of this material, and masks were strung across the x-direction as shown in Fig. 3. The x-direction measures approximately 22 ft, the y-direction 14 ft, and the floor-to-ceiling is 10 ft.

The centerline distance in the y-direction is approximately 7 ft (a conservative value of 8 ft was used in the analytical calculation). The lines were strung such that some of the lines/masks were closer to the front wall while others were by default closer to the back wall, but the masks were spaced to ensure that there was no shadowing. Before any masks were hung on the lines and irradiated, a photometer (model PMA2100; Solar Light Company, LLC) was used to measure the exposure in the sterilization room at nine different locations (designated with the stars in Fig. 2). The photometer was connected to a germicidal UVC detector (model PMA2122-WP; Solar Light Company, LLC) which has a range of 0–2000 µW/cm^2^ and a sensitivity to 254 nm wavelength, and both devices came with certificates of calibration^12^. These locations encompassed the limiting positions where masks would be placed, and two measurements were taken at each location. For one measurement the photometer window would face the front wall, and for the second measurement the photometer would face the back wall. This would ensure that both sides of the masks were receiving the necessary exposure, since the UVC radiation will not penetrate from one side of a mask through to the other side.

All measurements were taken by an individual wearing proper protective equipment. The individual would hold the photometer in position until the readout stabilized, and these values were recorded. The photometer readout is given in units of micro watts per cm^2^, an energy rate, but this can easily be converted into mJ/cm^2^, total energy. The minimum measurement was 100 µW/cm^2^, and this was observed at the position designated with the purple star in Fig. 2 with the photometer window facing the door. With an energy deposition rate of 100 µW/cm^2^, it will take 10 min to reach the exposure goal of 60 mJ/cm^2^. Since all other position readings were significantly larger than this, with a maximum of 300 µW/cm^2^, all mask positions were ensured to receive the appropriate exposure over a 10-min irradiation time. In fact, the three-bulb analytical solution of 7.65 min calculated above corresponds to a photometer reading of about 130 µW/cm^2^, which again, validates the chosen 10-min irradiation time.

### Simulation results

**Figure 4:**
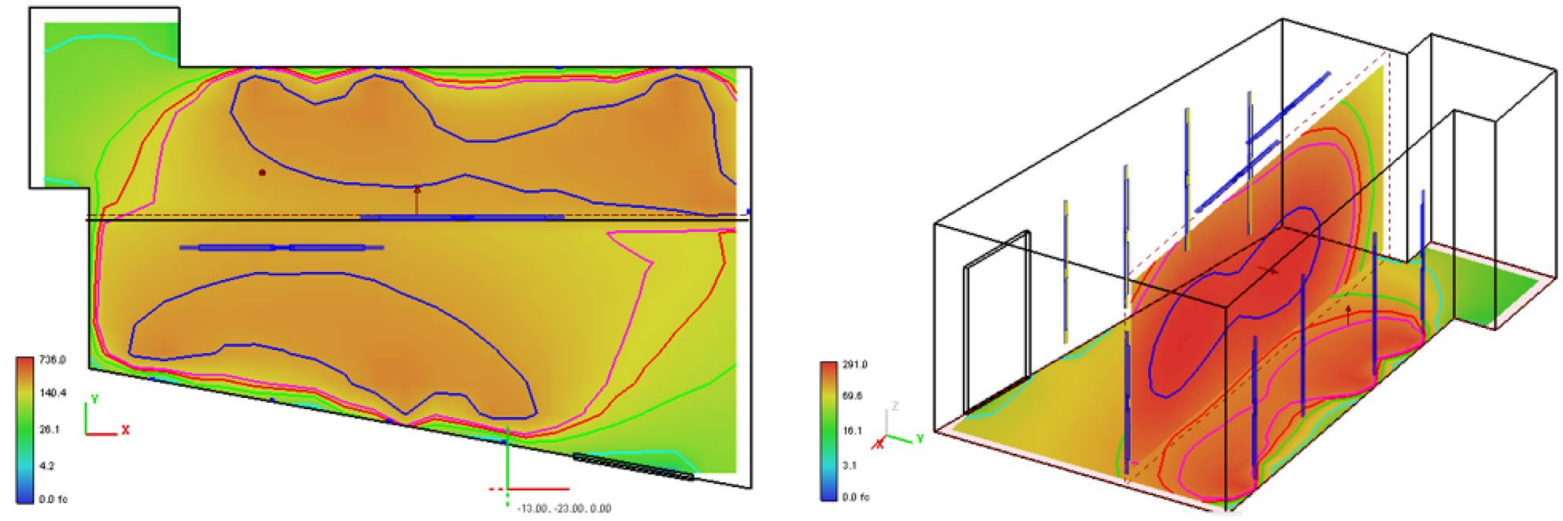
UV power density on the floor (left) and through a vertical plane spanning the center of the room (right).

Visual Lighting 2017 by Acuity Brands^13^ was used to simulate the light distribution inside the UV sterilization room. Visual is a software simulation tool commonly used in the commercial lighting design industry that enables the simulation of light reflectance and transmittance in a three-dimensional environment. The tool calculates lighting power density on various surfaces and the lighting transmittance through a plane. To perform the calculations, a model was developed to match the physical environment accounting both for the actual dimensions and the added reflectivity due to the aluminum foil on the walls. Lighting fixtures, or luminaires, are simulated using industry-standard .IES files. These files simulate the lighting lumen output and ray distribution unique to a particular luminaire. The actual lights used are Phillips TUV 64T5 HO 4P SE UNP/32. As these are bare lamps and not part of a specific luminaire, no IES file was available. Thus, an IES file for a single-lamp fluorescent luminaire was used, and the lumen output was adjusted to match the output of the Phillips lamps. Results are shown schematically in Fig. 4 with the reflectivity of the walls and ceiling set to 50%. This conservative value was chosen since the transmissivity of opaque solids is generally zero[^3^, p. 769] and the absorptivity of aluminum foil is approximately 0.15[^3^, p. 777]. Thus, the actual reflectivity of aluminum foil is closer to 85%.

The software natively performs calculations in foot-candles (fc) or lux (lx), and one fc is equal to approximately 1.57 µW/cm^2^. Thus, the minimum photometer reading of 100 µW/cm^2^ corresponds to 63.7 fc. As can be seen in Fig. 4 (left picture), the entire volume of the room encompassing the masks is subjected to well over 100 fc as designated with the yellow, orange, and red coloration. The red coloration in the right picture verifies that the masks located at the y-direction midplane receive upwards of 290 fc, which again validates that the masks will receive an exposure well above the minimum goal of 60 mJ/cm^2^.

## Quality assurance procedure

### Data collection

Upon launching the UVGI process, Institutional Review Board approval was obtained to collect two types of data, electronic UVGI associate data log and employee survey. The electronic UVGI associate data log included the following per cycle: number of N95s received for cleaning, number of N95s passing visual inspection for cleaning, cycle duration, number of masks decontaminated and number passing visual inspection post UVGI including mask integrity (including strap elasticity nose foam intact), and amount of UVC light frequency and duration. The employee 25- item survey was created by the researchers and administered to employees’ wearing his/her N95 mask post UVGI process. Survey questions included job title, years of experience, and length of time in current job. Additional questions included training, mask integrity, noticeable mask odor, and general wear questions associated to prior use of a N95 mask. A cover letter consent along with the survey was administered to employees at the end of their shift, both day and night shift. Descriptive statistics using Statistical Package for the Social Science, version 26.0 software (SPSS Inc., Chicago, IL) (IBM, 2014) was used for analyses.

Multiple steps were taken to ensure standardized workflows and processes were monitored for quality prior to the go-live and post-go live. Prior to go-live of the UVGI process, workflows defining the nurse obtaining a new or decontaminated N95 FFR through completion of UVGI decontamination were trialed numerous times as Sterile Processing staff were trained and demonstrated competency. Training and competency validation was completed over a one week. Workflow roles were created for the nurse, courier 1 (pick up contaminated N95 FFR at unit/department level and place in UVGI staging area), courier 2 (transport contaminated mask to UVGI staging area, place labeled bags on staging table, transport cart and crates for STERIS cleaning cycle), courier 3 (pick up clean cart and crates and transport to decontaminated area for loading of decontaminated N95 FFRs), courier 4 (Pick up contaminated N95 FFRs from surgical services and when decontaminated returned to surgical services), and the UVGI Associate (process steps from receiving to decontaminating the N95 FFRs to notifying courier for pick up to transport back to unit/department. An identification process was created and included labeling the N95 by the owner with first initial, last name, and employee identification number and place in a paper bag when removing the FFR during the shift and at the end of the shift placing it in a paper bag with the same information on the exterior of the bag.

Additionally, quality assurance (QA) steps were conducted prior to going live to ensure both qualitative and quantitative degradation had not occurred. A cohort of N95 FFRs were decontaminated and used in standardized quantitative fit testing to ensure the integrity of the N95 FFR was maintained over five decontamination cycles. The UVC quality control testing measuring the UVC light intensity was conducted each cycle (via UVC sensor). The UVC light frequency and duration were recorded to ensure the required disinfection photometer reading of 70 (or greater) 70 µW/cm^2^ for 10 min was reached. This data was collected with each N95 FFR both pre-and post-implementation of the UVGI decontamination process.

A qualitative smell test was completed by two individuals to determine if there were any noticeable odors. None were detected. After aeration cycle, the N95 FFRs underwent a QA process to ensure that there was no physical or performance degradation. The decontaminated N95 fit testing process secured optimal performance and were tested on two individuals with contradictory facial structures to ensure no loss of fit or seal.

### QA results

The final sample included 78 participants. Most were Black/African American (45%), females (82%), and Registered Nurses (46%) with an average 10 years of experience in current job. The demographics are listed in Table 1 with both the numeric and percent values.

**Table 1:** Participant demographics.

Characteristics	Total (*N* = 78)
Age, M (SD)	41.47 (12.81)
Female, n (%)	63 (81.8)
**Ethnicity, n (%)**	
White/Caucasian	32 (42.1)
Black/African American	34 (44.7)
Asian or Pacific Islander	4 (5.3)
Hispanic/Latino	4 (5.3)
Other	2 (2.6)
**Job title, n (%)**	
Registered nurse (RN)	35 (45.5)
Care partner (CP)	12 (15.6)
Sterile processing tech	9 (11.7)
Respiratory therapist (RT)	11(14.3)
Other	9 (11.7)
Years’ experience in current job, M (SD)	10.2 (9.7)

In the last year, all participants reported being trained on proper donning/doffing of various N95 face masks, and these numbers are given in Table 2. The majority of N95 masks decontaminated were 3 M 1860s. A majority (80%) of participants reported being familiar with UVGI decontamination and 72% perceived implementing UVGI mitigated shortages.

**Table 2:** N95 FFR characteristics.

Characteristics	Total (*N* = 78)
Training on proper use (donning/doffing) of FFR in the last year, yes, n (%)	78 (100)
**N95 FFR, n (%)**	
3M 1860	33 (43.4)
3M 1860S	20 (26.3)
3M 1805	4 (5.3)
8210	4 (5.3)
Other	9 (19.7)
Familiar UVGI decontamination/reuse, yes, n (%)	64 (82.1)
Implementing UVGI mitigated N-95 shortages, yes, n (%)	56 (71.8)

On average masks were decontaminated 3.5 times and discarded on average once after decontamination. Most common concerns reported with N95 masks after UVGI included nose foam which included flat, harder to form to face, loose fitting; mask durability strap integrity, shape softer not as firm; and odor which included burn and chemical smell. Table 3 summarizes the survey results.

**Table 3:** UVGI survey findings.

Characteristics	Total (*N* = 78)
Reused N95, M (SD)	3.5 (3.2)
Discarded N95 after UVGI, M (SD)	1.0 (.31)
**N95 concerns after UVGI, M (SD)**	
Nose foam	1.7 (.45)
Durability	1.5 (.49)
Odor	1.5 (.50)
Seal (fit check) successful throughout shift worked, no, n (%)	15 (19.2)
N-95 durability concerns, no, n (%)	49 (62.8)
Concerns with nose foam, no, n (%)	57 (73.1)
Concerns about ear straps, no, n (%)	68 (87.1)
Concerns about odor, no, n (%)	46 (50.0)

A total of 434 decontamination cycles were performed at the hospital between April 27, 2020, and November 17, 2020, with zero fail loads/runs. This amounted to a total of 8983 sterilized masks.

## Conclusions

Prior to COVID-19, N95 mask were worn and discarded after daily use. At the onset of COVID-19, supply and demand variation became difficult to predict as hospitalizations quickly escalated and suppliers of N95 masks faced a lag with manufacturers production to meet the needs across the country. It was evident early in the pandemic that strategic and innovative processes needed to be developed and implemented to ensure adequate numbers of N95 mask were available to support safety of all caregivers.

Using UVGI to decontaminate N95 masks was an effective and efficient process to ensure safety of mask reuse. Workflow to ensure standardization of processes and measurement of irradiation was critical to ensure sustainability. Workflows ranged from pickup of contaminated N95s, UVGI irradiation process, to delivery of irradiated N95s for use for day and night shifts by unit or department. Caregivers received their irradiated N95 mask back based on unique identifiers.

The use of UVGI to decontaminate N95 masks successfully supported caregivers ability to provide care to patients while maintaining their personal safety. Staff perceptions of efficacy related to UVGI irradiation and N95 reuse s was positive. Caregiver feedback indicated that greater than 60% expressed no concern around durability of decontaminated masks. Quality checks ensured all compromised masks were disposed of and never returned to a caregiver. Greater than 70% of caregivers had no concerns with the nose foam and greater than 85% had no concerns about the ear straps that impacted the fit test for each time used. Ear straps were the greatest reason for disposing of N95 masks. Concern for an odor associated with UVGI irradiation was noted by half of caregiver respondents. UVGI irradiation of N95 masks ran multiple loads daily for 205 consecutive days with zero failed loads throughout the process.

Assuring the UVGI irradiation process was successful required a collaborative partnership with content experts from academia and healthcare. Evidence grounded in engineering, nursing, medicine, and quality practices provided the foundation for the UVGI process implemented and sustained. As supply and demand for N95 mask evolved to a more predictable pattern, the UVGI irradiation process was reduced, ending on day 205. Academic and experiential knowledge gained during the UVGI project has been invaluable and will support the organization to reactivate quickly if needed in the future.

### Ethical statement

All methods were carried out in accordance with relevant guidelines and regulations. The experimental protocol, likewise, was approved by the Wellstar Institutional Review Board. Informed consent was obtained from all participants, or if participants were under 18, from a parent and/or legal guardian. This is indicated in the attached Wellstar Institutional Review Board letter.
